# Simultaneous detection of ovine and caprine DNA in meat and dairy products using triplex *Taq*Man real‐time PCR

**DOI:** 10.1002/fsn3.1936

**Published:** 2020-10-19

**Authors:** Liang Guo, Yuan Yu, Wei‐Liang Xu, Chun‐Dong Li, Guo‐Qiang Liu, Lemuge Qi, Jian‐Xing Luo, Yuan‐Sheng Guo

**Affiliations:** ^1^ Xilin Gol Institute of Bioengineering Xilin Gol Food Testing and Risk Assessment Center Xilingol Vocational College Xilinhot China

**Keywords:** authentication, caprine, ovine, *Taq*Man, triplex

## Abstract

In this study, we report a new approach for the detection of ovine and caprine DNA in meat and dairy products using real‐time PCR protocol. Our new approach is based on the use of endogenous control and species‐specific *Taq*Man fluorescence probes. With this methodology, we specifically detected ovine and caprine DNA in meat and dairy products, with limits of detection of 0.001 ng and 0.01 ng for fresh and processed ovine meats, respectively, and 0.00025 ng, 0.005 ng, and 0.01 ng for caprine meat, milk, and cheese, respectively. Artificial meat and milk mixtures from sheep and goat were used to validate the protocol. Our results support that *Taq*Man real‐time PCR with endogenous control is an efficient and accurate method to detect DNA from sheep and goat in meat and dairy products.

## Graphical abstract

Our triplex real‐time PCR method with endogenous control is a specific and highly sensitive technique for the certification of meat and dairy products derived from sheep and goat.

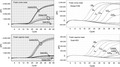

## 1 INTRODUCTION

Meat and dairy products provide essential nutrients that support growth, functions, immunity, and general human well‐being. Xilingol grassland (203,000 km^2^) in China is a natural grazing region, known for the free raising of animals for human consumption and its low pollution levels, resulting in the production of high‐quality meat and dairy products from sheep and goat, among others. The average annual output of sheep and goat in Xilingol grassland is more than ten million of animals. Nevertheless, some producers and traders deliberately adulterate meat and dairy products with less expensive substitutes, which unbalances market competition and disregards consumer's interests. Further, product authentication is required to manage health risks and to comply with religious norms. Therefore, the development of efficient approaches for product authentication is essential to ensure the purity of food products. Here, we developed a specific and highly sensitive method to evaluate adulteration of meat and milk from sheep and goat based on DNA detection using real‐time PCR with *Taq*Man technology.

Distinct analytical PCR‐based methods have been developed for DNA detection in animal‐derived products such as meat and dairy (Abbas et al., 2018; Bohme et al., 2019; Lo & Shaw, 2018). Conventional PCR (Fajardo et al., 2006; Kumar et al., 2014; Pfeiffer et al., 2004), multiplex PCR (Golinelli et al., 2014; Safdar & Junejo, 2015; Xue et al., 2017), conventional real‐time PCR (Li et al., 2019; López‐Calleja et al., 2007; Tanabe et al., 2007), and multiplex real‐time PCR (Agrimonti et al., 2015; Di Domenico et al., 2017; Guo et al., 2019) are standard techniques used in the detection of ovine and caprine DNA. Here, we developed a multiplex real‐time PCR with endogenous control for simultaneous detection of ovine and caprine DNA in meat and dairy products. Multiplex real‐time PCR is more efficient and less time‐consuming than conventional real‐time PCR and PCR due to lack of electrophoresis and simultaneous amplification of different target genes. Furthermore, the simultaneous amplification of an endogenous control avoids false‐negative results.

## 2 MATERIALS AND METHODS

### 2.1 Sample preparation and DNA isolation

Fresh and processed meat samples from sheep, goat, beef, horse, pork, chicken, duck, goose, dog, rabbit, cat, and fish were purchased from 109 supermarkets and DKL shopping mall in Xilingol region in Inner Mongolia. Milk from goat, cow, and mare, as well as koumiss, was obtained from Plain Mountain Pasture from Xilingol region. Caprine cheese (Queserias Entrepinares) was purchased from the Shanghai Rongyue company, and bovine cheese was obtained from Ximulike dairy company from Xilingol region.

DNA from meat and dairy products was isolated with TaKaRa MiniBEST Universal Genomic DNA Extraction Kit, using a modified CTAB method (Guo et al., 2018). DNA concentration and quality were assessed using a NanoDrop 2000c at a wavelength of 260 nm and 280 nm.

### 2.2 Oligonucleotide development and reaction settings

One species‐conserved primer pair (LP1 and RP1) and endogenous control (control probe) as well as species‐specific probes (ovine probe and caprine probe) of oligonucleotides for sheep, goat, and endogenous control, respectively, were designed to allow simultaneous amplification by triplex real‐time PCR. All oligonucleotides were designed with Primer5 software, to target highly conserved regions of mitochondrial 12S ribosomal DNA. The accession numbers for the target sequences were KR868678.1, KP981380.1, KF938336.1, KF938327.1, KF938319.1, KY305183.1, KP273589.1, KP271023.1, KP662716.1, and KP677509.1. Oligonucleotides were synthesized and purified using HPLC by Ruibiotech company. A list of primers and probes is shown in Table 1. The innovation of design is to guarantee the simultaneous triplex real‐time PCR with endogenous control in the single PCR. The triplex real‐time PCR shares one identical forward primer (LP1) and two reverse primers (Ovine‐RP1 and Caprine‐RP1) with one different base, and control probe can anneal the same targeted amplified sequence with ovine probe and caprine probe in the triplex real‐time PCR.

**TABLE 1:** *Taq*Man real‐time PCR primers and probes

Primer and probe	Sequence (5′ to 3′)
LP1	TTGAATCAGGCCATGAAGC
Ovine‐RP1	CTTACCTTGTTACGACTTGTCTC
Caprine‐RP1	CTTACCTTGTTACGACTTATCTC
Ovine probe	FAM‐CCTCTCGTGTGGTTGATATATGTAAATAGGTT‐TAMRA
Caprine probe	HEX‐TCTCATGTAGTTGATGCGTGTTAATAGGCT‐TAMRA
Control probe	ROX‐ACACACCGCCCGTCACCCT‐BHQ‐2

The triplex real‐time PCR mixture (20 μl) was composed as follows: 10 μl Probe qPCR SuperMix (TransGen), 1 μl LP1 (10 μmol/L), 0.5 μl Ovine‐RP1 (10 μmol/L), 0.5 μl Caprine‐RP1 (10 μmol/L), 1 μl ovine probe (10 μmol/L), 1 μl caprine probe (10 μmol/L), 1 μl control probe (10 μmol/L), 1 μl template (100 ng/μl), and 4 μl ddH_2_O. PCRs were performed in an ABI 7300 plus thermocycler (Applied Biosystems), with the following program: 94°C for 30 s followed by 40 cycles of 94°C for 5 s and 60°C for 31 s.

### 2.3 Reaction specificity and sensitivity evaluation

Samples of raw meat from sheep, goat, beef, horse, pork, chicken, duck, goose, dog, rabbit, cat, and fish were used to validate our newly designed primers and probes. Ct values were calculated for eight different types of fresh ovine and caprine meats and four types of processed ovine meats, and three types of milk and three types of cheese from goat were utilized to verify the specificity.

Sensitivity of the method was evaluated by calculating the limit of detection (LOD). LOD values were determined using 10‐fold and twofold serial dilutions of DNA from meat and dairy products (100, 10, 1, 0.1, 0.01, 0.005, 0.0025, 0.001, 0.0005, 0.00025, 0.0001, 0.00005, 0.000025, and 0.00001 ng/μl). Twenty replicates per dilution were used, and the LOD was analyzed by Probit analysis (Finney, 1971).

## 3 RESULTS AND DISCUSSION

### 3.1 Specificity of triplex real‐time PCR in the amplification of DNA from meat and dairy

**FIGURE 1:**
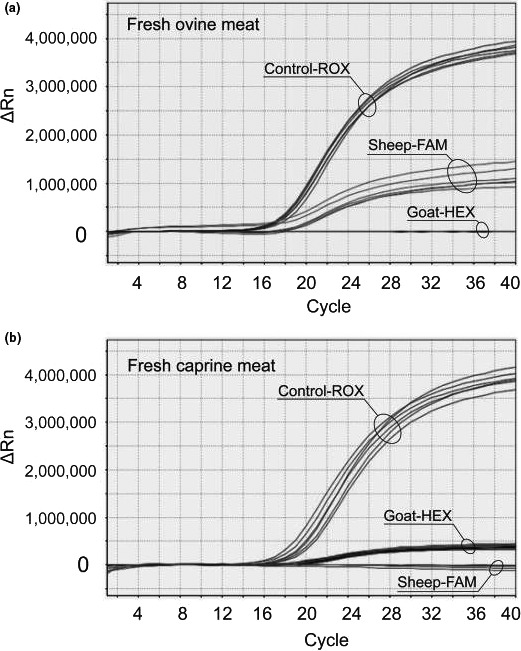
Triplex real‐time PCR amplification plots for the detection of DNA from sheep (a) and goat (b) from fresh meat samples

In the *Taq*Man real‐time PCR assay, the ovine‐specific probe was labeled with FAM (fluorophore) and TAMRA (quencher). The caprine‐specific probe was labeled with HEX and TAMRA. The endogenous control probe was labeled with ROX and BHQ‐2. The use of different fluorophores (FAM, HEX, and ROX) allowed the simultaneous detection of three distinct fluorescence signals, thus allowing the identification of DNA from different sources (in this case, from sheep, goat, and the endogenous control). Figure 1 shows the amplification plots of triplex real‐time PCR using DNA from fresh ovine and caprine meat as template. An endogenous control (Control‐ROX) was also amplified to eliminate false‐negative results. As shown in Figure 1, amplification plots show distinct profiles, which translate the specificity of each amplification reaction. Ct values relative to the amplification of 8 independent samples of ovine and caprine DNA (3 replicates per sample) as well as to the amplification of meat‐derived DNA from additional species (10 different species, 3 replicates per taxon) are shown in Table 2. The results show that the amplification of sheep‐ and goat‐derived DNA was steadily showed. Furthermore, no amplification was observed when nontarget animal DNA was used as a template for the PCRs, supporting the specificity of the method. The above data illustrated that the triplex real‐time PCR with the endogenous control was specific for the simultaneous detection of ovine and caprine DNA isolated from the respective fresh meat.

**TABLE 2:** The Ct values in the real‐time PCR assay for the ovine and caprine detection in the fresh meats

Samples	Ct value^a^		
	Sheep‐FAM	Goat‐HEX	Control‐ROX
Sheep 1	20.45 ± 1.53	0	18.06 ± 0.09
Sheep 2	20.91 ± 1.12	0	18.53 ± 0.25
Sheep 3	20.38 ± 0.03	0	20.87 ± 0.28
Sheep 4	20.97 ± 0.34	0	20.89 ± 0.29
Sheep 5	24.06 ± 2.76	0	24.05 ± 0.73
Sheep 6	27.08 ± 2.69	0	24.51 ± 0.45
Sheep 7	21.05 ± 0.83	0	18.43 ± 0.14
Sheep 8	18.28 ± 0.75	0	18.44 ± 0.91
Goat 1	0	17.2 ± 0.16	17.77 ± 0.72
Goat 2	0	17.95 ± 0.46	17.95 ± 1.43
Goat 3	0	21.09 ± 0.78	19.28 ± 0.87
Goat 4	0	21.23 ± 1.04	19.52 ± 0.7
Goat 5	0	24.69 ± 0.12	25.46 ± 0.35
Goat 6	0	26.34 ± 1.42	26.6 ± 1.08
Goat 7	0	22.85 ± 0.6	23.27 ± 0.19
Goat 8	0	22.91 ± 1.13	23.48 ± 1.14
Beef	0	0	18.52 ± 0.12
Horse	0	0	21.48 ± 0.41
Pork	0	0	19.02 ± 0.49
Chicken	0	0	N/A
Duck	0	0	N/A
Goose	0	0	N/A
Dog	0	0	N/A
Rabbit	0	0	N/A
Cat	0	0	N/A
Fish	0	0	N/A

Abbreviation: N/A, not applicable.

^a^ Data (average ± *SD*) represent three replicates.

**FIGURE 2:**
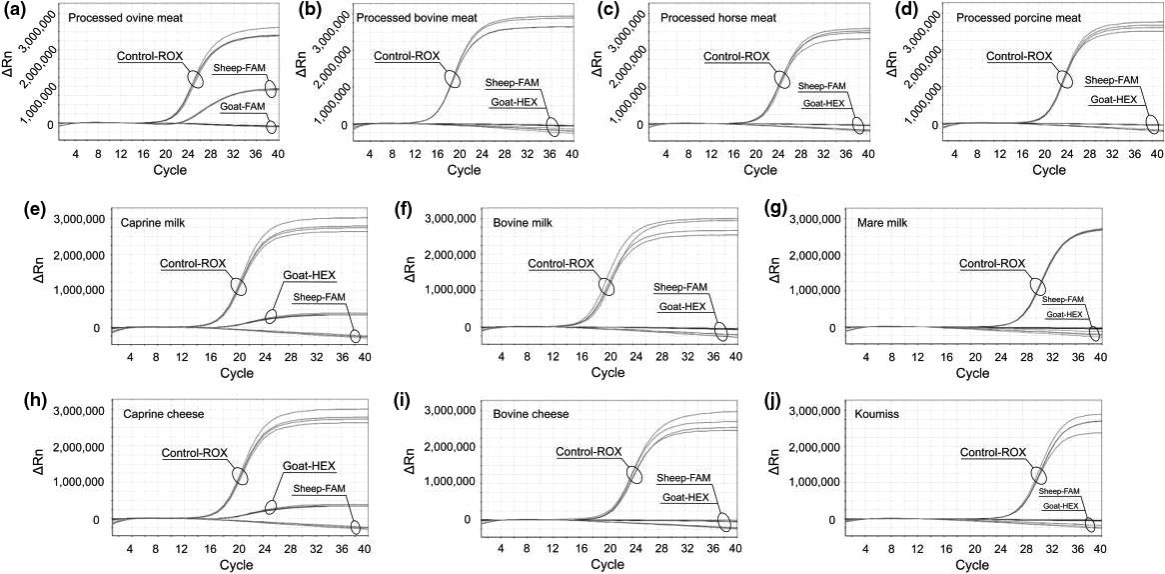
Triplex real‐time PCR amplification plots for the identification of DNA from processed ovine meat (a), processed bovine meat (b), processed horse meat (c), processed porcine meat (d), caprine milk (e), bovine milk (f), mare milk (g), caprine cheese (h), bovine cheese (i), and koumiss (j)

Compared with fresh meat, processed meat products are more likely to be adulterated due to physical processing. Processed meat samples from sheep, cattle, horse, and pig were used to evaluate the specificity of the method, and the corresponding plots show that amplification was specific to ovine and caprine samples, whereas no amplification was observed in samples from other species, using our sheep‐ and goat‐specific primers (Figure 2a‐d). Corresponding Ct values are shown in Table 3 (processed ovine meat: 4 independent samples per assay, 3 replicates per sample; other species: 2 independent samples per taxon, 3 replicates). The Ct values of the processed ovine meats identified by Sheep‐FAM were steadily showed, and no amplification was obtained with the DNA of nontarget species. Thus, our data show that triplex real‐time PCR with an endogenous control is specific for the simultaneous detection ovine and caprine DNA in processed meat products.

**TABLE 3:** The Ct values in the triplex real‐time PCR assay for the ovine and caprine identification in the processed meats and dairy products

Samples	Ct value^a^		
	Sheep‐FAM	Goat‐HEX	Control‐ROX
Processed ovine meat 1	14.06 ± 1.47	0	22.29 ± 0.34
Processed ovine meat 2	15.02 ± 0.05	0	22.46 ± 0.19
Processed ovine meat 3	17.42 ± 0.64	0	25.07 ± 0.17
Processed ovine meat 4	16.43 ± 0.76	0	25 ± 0.04
Processed bovine meat 1	0	0	14.17 ± 0.04
Processed bovine meat 2	0	0	14.12 ± 0.22
Processed horse meat 1	0	0	18.85 ± 0.08
Processed horse meat 2	0	0	21.06 ± 0.32
Processed porcine meat 1	0	0	17.03 ± 0.28
Processed porcine meat 2	0	0	16.38 ± 0.27
Caprine milk 1	0	15.16 ± 0.34	16.69 ± 0.23
Caprine milk 2	0	15.88 ± 0.41	16.64 ± 1.54
Caprine milk 3	0	15.06 ± 0.58	16.21 ± 0.68
Bovine milk 1	0	0	14.75 ± 0.34
Bovine milk 2	0	0	15.27 ± 0.35
Horse milk 1	0	0	25.34 ± 0.08
Horse milk 2	0	0	25.88 ± 0.40
Caprine cheese 1	0	20.54 ± 0.27	22.07 ± 0.74
Caprine cheese 2	0	20.43 ± 0.09	21.85 ± 0.47
Caprine cheese 3	0	20.28 ± 0.07	21.54 ± 0.15
Bovine cheese 1	0	0	18.68 ± 0.13
Bovine cheese 2	0	0	19.05 ± 0.29
Koumiss 1	0	0	20.86 ± 0.81
Koumiss 2	0	0	18.67 ± 0.92

Abbreviation: N/A, not applicable.

^a^ Data (average ± *SD*) represent three replicates.

Sheep and goat are important livestock in Xilingol grassland, being mainly raised for meat, milk, and fleece production. We then evaluated the specificity of our method in DNA isolated from caprine milk and cheese. Figure 2e‐h show specific amplification of Goat‐HEX, whereas Control‐ROX was amplified to indicate the reaction of real‐time PCR for eliminating false‐negative results. DNA from bovine (Figure 2f) and mare milk (Figure 2g), as well as from bovine cheese (Figure 2i) and koumiss (Figure 2j), was used as the negative control. The Ct values (average ± *SD*) of caprine milk, bovine milk, mare milk, caprine cheese, bovine cheese, and koumiss are illustrated in Table 3. The above data demonstrate that our triplex real‐time PCR is a specific technique for the authentication of caprine milk and cheese products.

Generally, the identification of DNA from meat and dairy products is based on conventional PCR and real‐time PCR with fluorescent dye. However, when compared to *Taq*Man‐based methods, conventional PCR shows limited specificity and is more time‐consuming, as it only allows the identification of a single species in each reaction. Our multiplex real‐time PCR was designed to detect several DNA species in a single PCR using species‐specific *Taq*Man probes. For that, it is fundamental to design specific primers and probes that are compatible in the same reaction. In the context of the authentication of ovine and caprine meat and dairy products, this type of approach has not been used, so far. Our results show that our triplex real‐time PCR is suitable for the specific identification of ovine and caprine DNA, both in fresh and in processed meat products, as well as in caprine milk and dairy products. The simultaneous detection of DNA from different species contributes to lowering reagent cost, consumables, and reduces experimental time by half due to the addition of a species‐specific probe. Furthermore, the performance of the PCR can be monitored by the inclusion of the endogenous control in the same reaction of the target template, thus avoiding false‐negative results.

### 3.2 Sensitivity of triplex real‐time PCR in the amplification of DNA from meat and dairy products

**FIGURE 3:**
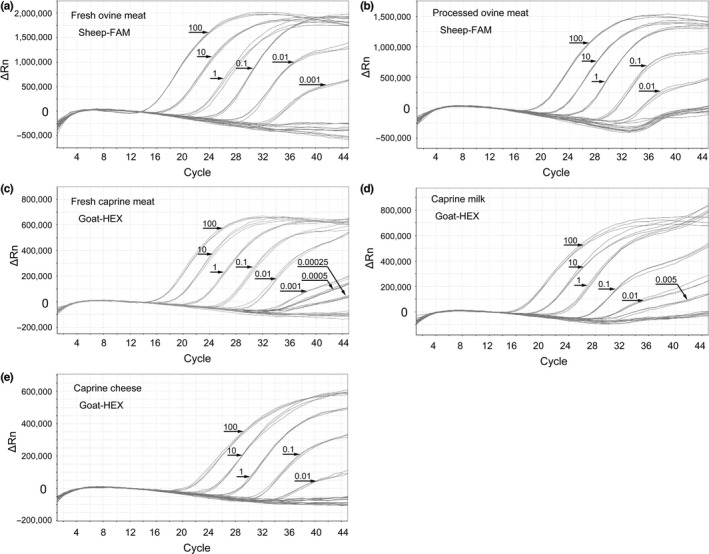
Real‐time PCR amplification plots for the gradient dilution of DNA extracted from fresh ovine meat (a), processed ovine meat (b), fresh caprine meat (c), caprine milk (d), and caprine cheese (e)

As shown in Figure 3a,b, the LOD of ovine DNA was of 0.001 ng (DNA isolated from fresh ovine meat) and of 0.01 ng (DNA isolated from processed ovine meat; confidence limit: 95%). Moreover, as shown in Figure 3c‐e, the LOD of caprine DNA identification was of 0.00025 ng (DNA isolated from fresh caprine meat) and of 0.005 ng and 0.01 ng (DNA isolated from caprine milk and cheese, respectively, confidence limit: 95%). As shown in Table 4, the Ct values (average ± *SD*) of the triplex real‐time PCR increased with increasing dilution of meat and dairy DNA. The above results show that the LOD of DNA derived from fresh meat and raw milk is lower than LOD of DNA derived from processed meat and cheese, which might be due to the higher integrity of the DNA from the first two. Thus, the LOD results illustrated that the real‐time PCR based on designed primers and probes were sensitive to identify the target DNA in the meat and dairy products originated from sheep and goat. The LODs observed in the triplex real‐time PCR for detection of ovine and caprine DNA were similar to other PCR‐based methods (Di Domenico et al., 2017; Guo et al., 2019; Xue et al., 2017). These results demonstrate that our newly developed triplex real‐time PCR is sensitive in the detection of ovine and caprine DNA in meat and dairy products.

**TABLE 4:** The Ct values in the real‐time PCR assay for the sensitivity of identification of meat and dairy products from sheep and goat

Samples	Input DNA amount (ng)	Ct value^a^	
		Sheep‐FAM	Goat‐HEX
Fresh ovine meat	100	14.24 ± 0.21	N/A
	10	17.13 ± 0.35	N/A
	1	21.82 ± 0.96	N/A
	0.1	25.99 ± 0.58	N/A
	0.01	30.40 ± 0.49	N/A
	0.001	35.34 ± 0.90	N/A
	0.0005	0	N/A
	0.00025	0	N/A
	0.0001	0	N/A
	0.00001	0	N/A
Processed ovine meat	100	19.24 ± 0.49	N/A
	10	23.15 ± 0.68	N/A
	1	26.80 ± 1.11	N/A
	0.1	31.32 ± 0.65	N/A
	0.01	36.21 ± 1.10	N/A
	0.005	0	N/A
	0.0025	0	N/A
	0.001	0	N/A
	0.0001	0	N/A
	0.00001	0	N/A
Fresh caprine meat	100	N/A	14.69 ± 0.32
	10	N/A	17.61 ± 0.41
	1	N/A	21.3 ± 2.15
	0.1	N/A	26.03 ± 0.61
	0.01	N/A	30.58 ± 0.55
	0.001	N/A	34.62 ± 1.07
	0.0005	N/A	34.96 ± 1.86
	0.00025	N/A	37.01 ± 1.3
	0.0001	N/A	0
	0.00001	N/A	0
Caprine milk	100	N/A	15.03 ± 0.31
	10	N/A	19.08 ± 0.28
	1	N/A	23.23 ± 0.49
	0.1	N/A	27.36 ± 0.59
	0.01	N/A	32.77 ± 1.13
	0.005	N/A	34.55 ± 1.06
	0.0025	N/A	0
	0.001	N/A	0
	0.0001	N/A	0
	0.00001	N/A	0
Caprine cheese	100	N/A	20.95 ± 0.55
	10	N/A	24.86 ± 0.50
	1	N/A	28.88 ± 0.41
	0.1	N/A	33.79 ± 1.33
	0.01	N/A	37.65 ± 1.06
	0.005	N/A	0
	0.0025	N/A	0
	0.001	N/A	0
	0.0001	N/A	0
	0.00001	N/A	0

Abbreviation: N/A, not applicable.

^a^ Data (average ± *SD*) represent 20 replicates.

**FIGURE 4:**
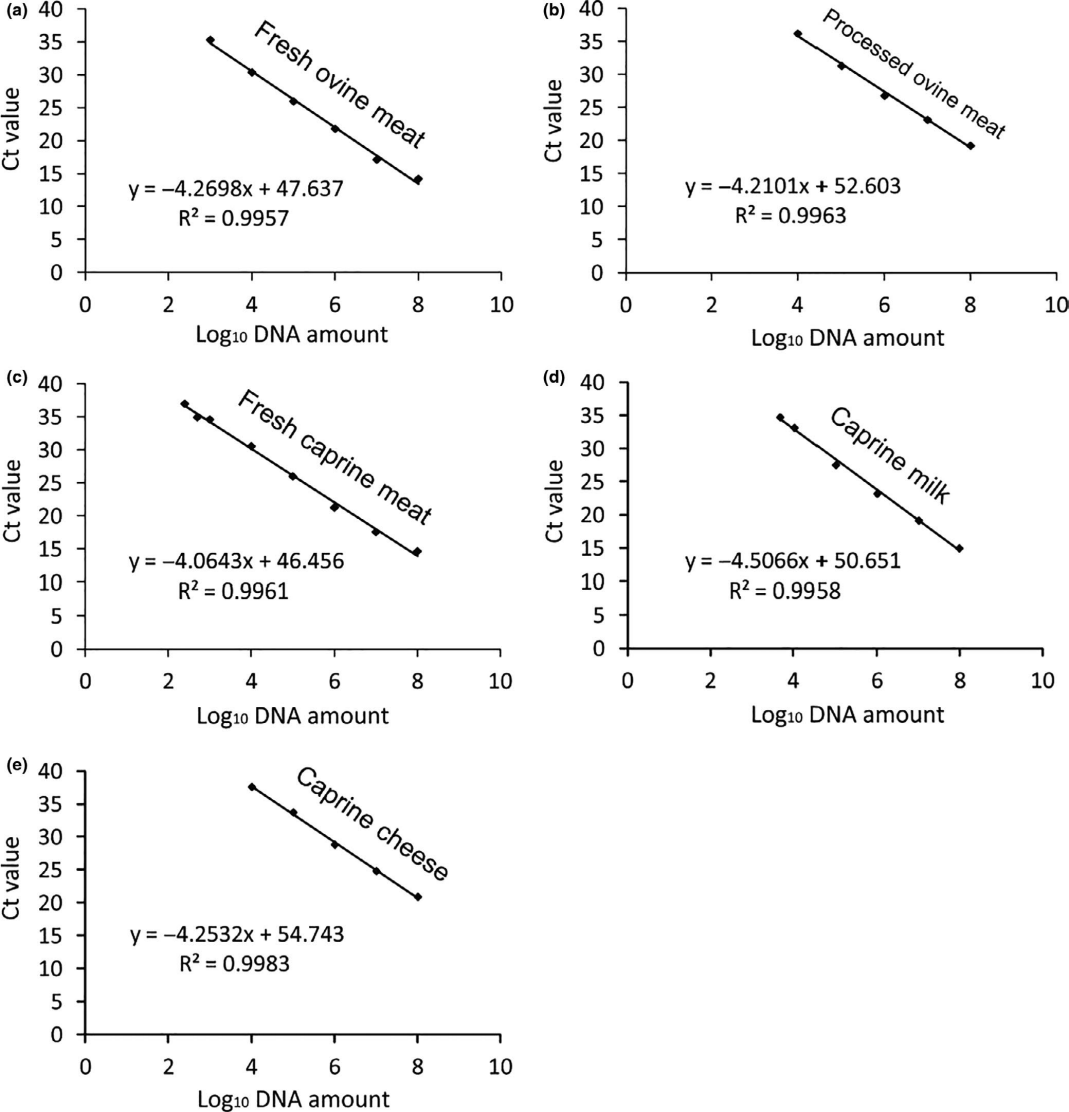
Calibration curves for the quantification of ovine and caprine DNA in fresh ovine meat (a), processed ovine meat (b), fresh caprine meat (c), caprine milk (d), and caprine cheese (e)

As shown in Figure 4, the calibration curves were constructed by plotting the Ct values versus the logarithm of DNA concentration in solution. The calibration curve was determined with 20 replicate analyses. The slopes of the calibration curves were determined as follows: −4.2698 for fresh ovine meat DNA (Figure 4a), −4.2101 for processed ovine meat DNA (Figure 4b), −4.0643 for fresh caprine meat DNA (Figure 4c), −4.5066 for caprine milk DNA (Figure 4d), and −4.2532 for caprine cheese DNA (Figure 4e), with the corresponding correlation coefficients of 0.9957 (Figure 4a), 0.9963 (Figure 4b), 0.9961 (Figure 4c), 0.9958 (Figure 4d), and 0.9983 (Figure 4e). Thus, we conclude that our real‐time PCR method demonstrates good calibration linearity and is suitable for the quantification of ovine and caprine DNA in meat and dairy products.

### 3.3 Triplex real‐time PCR in DNA detection from artificial meat and dairy mixtures

**FIGURE 5:**
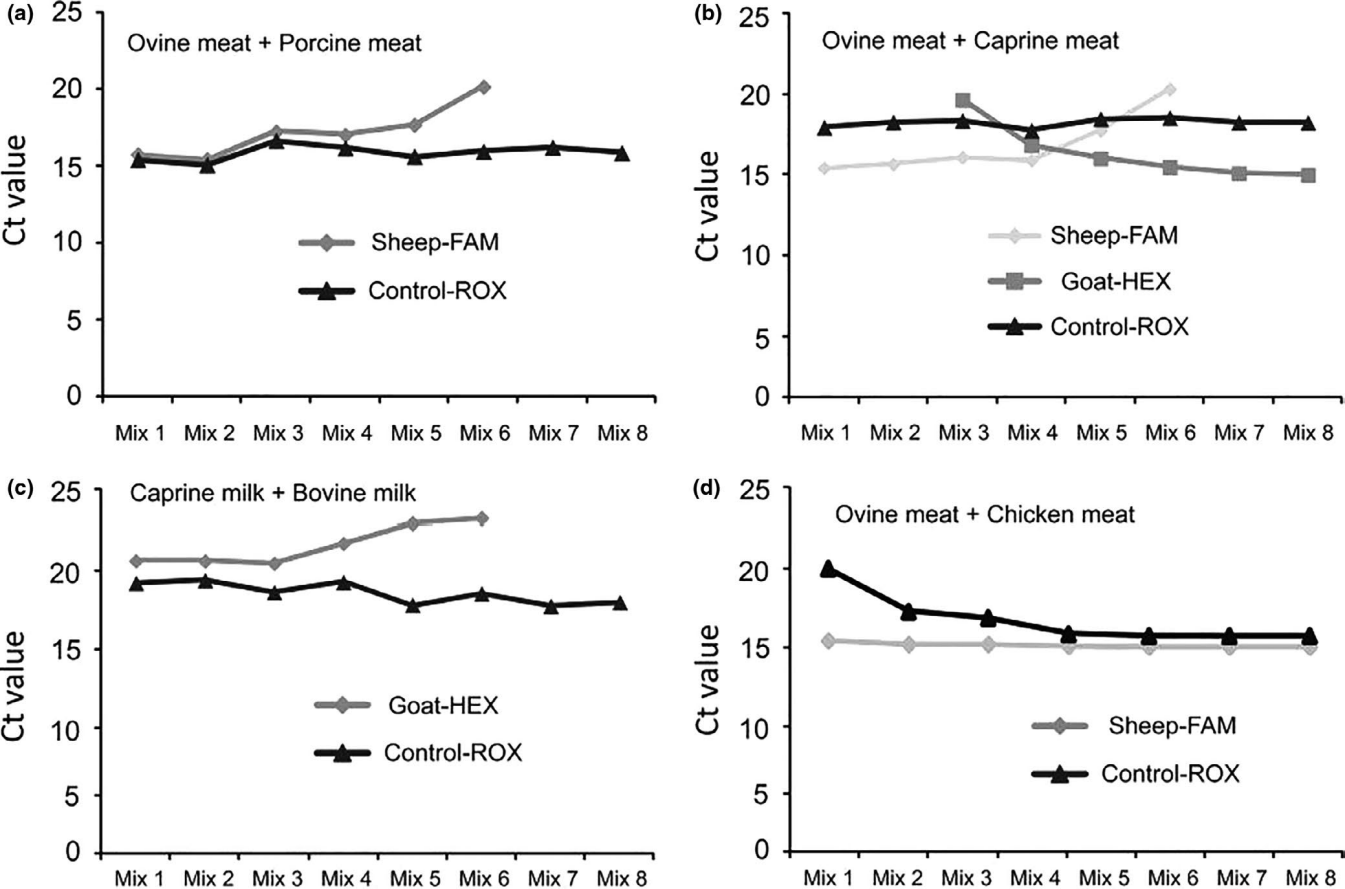
Triplex real‐time PCR assay for the identification of DNA from sheep and goat in artificial ovine and porcine meat mixtures (a), ovine and caprine meat mixtures (b), caprine and bovine milk mixtures (c), and ovine and chicken meat mixtures (d). The results were confirmed by 20 replicates

To evaluate the efficiency of the triplex real‐time PCR in the detection of DNA from meat mixtures, we isolated DNA from artificial binary meat mixtures, containing ovine and porcine meat, and used it as template for PCR. The percentages of ovine meat in the mixtures were 99.9%, 99%, 90%, 70%, 30%, 10%, 1%, and 0.1% (w/w). The corresponding percentages of porcine meat in the mixtures were 0.1%, 1%, 10%, 30%, 70%, 90%, 99%, and 99.9%. As shown in Figure 5a and Table 5, ovine DNA Ct values increased with the decrease of ovine meat percentage in the mixtures, with Ct = 0 for 1% and 0.1% ovine meat percentage.

**TABLE 5:** The Ct values in the triplex real‐time PCR for the meat and milk mixtures from sheep and goat

Samples	Volume (%)						Ct value^a^		
	Ovine meat	Porcine meat	Caprine meat	Chicken meat	Caprine milk	Bovine milk	Sheep‐FAM	Goat‐HEX	Control‐ROX
Mix 1	99.9	0.1	N/A	N/A	N/A	N/A	15.49 ± 0.42	0	15.58 ± 0.19
Mix 2	99	1	N/A	N/A	N/A	N/A	15.17 ± 0.29	0	15.25 ± 0.14
Mix 3	90	10	N/A	N/A	N/A	N/A	16.98 ± 0.36	0	16.83 ± 0.14
Mix 4	70	30	N/A	N/A	N/A	N/A	16.76 ± 0.42	0	16.35 ± 0.13
Mix 5	30	70	N/A	N/A	N/A	N/A	17.42 ± 0.23	0	15.79 ± 0.1
Mix 6	10	90	N/A	N/A	N/A	N/A	19.9 ± 0.53	0	16.12 ± 0.19
Mix 7	1	99	N/A	N/A	N/A	N/A	0	0	16.4 ± 0.15
Mix 8	0.1	99.9	N/A	N/A	N/A	N/A	0	0	16.03 ± 0.18
Mix 1	99.9	N/A	0.1	N/A	N/A	N/A	15.46 ± 0.19	0	17.67 ± 0.09
Mix 2	99	N/A	1	N/A	N/A	N/A	15.68 ± 0.16	0	18 ± 0.05
Mix 3	90	N/A	10	N/A	N/A	N/A	16.11 ± 0.34	19.69 ± 0.5	18.08 ± 0.08
Mix 4	70	N/A	30	N/A	N/A	N/A	15.93 ± 0.27	16.86 ± 0.36	17.48 ± 0.17
Mix 5	30	N/A	70	N/A	N/A	N/A	17.77 ± 1.3	16.05 ± 0.32	18.2 ± 0.08
Mix 6	10	N/A	90	N/A	N/A	N/A	20.34 ± 0.51	15.50 ± 0.2	18.27 ± 0.14
Mix 7	1	N/A	99	N/A	N/A	N/A	0	15.12 ± 0.19	17.98 ± 0.13
Mix 8	0.1	N/A	99.9	N/A	N/A	N/A	0	15.02 ± 0.21	17.97 ± 0.08
Mix 1	N/A	N/A	N/A	N/A	99	1	0	21.18 ± 0.32	19.78 ± 0.25
Mix 2	N/A	N/A	N/A	N/A	95	5	0	21.17 ± 0.4	19.94 ± 0.22
Mix 3	N/A	N/A	N/A	N/A	90	10	0	21.03 ± 0.48	19.19 ± 0.29
Mix 4	N/A	N/A	N/A	N/A	70	30	0	22.28 ± 0.45	19.83 ± 0.29
Mix 5	N/A	N/A	N/A	N/A	30	70	0	23.5 ± 0.44	18.39 ± 0.27
Mix 6	N/A	N/A	N/A	N/A	10	90	0	23.81 ± 0.21	19.11 ± 0.26
Mix 7	N/A	N/A	N/A	N/A	5	95	0	0	18.32 ± 0.23
Mix 8	N/A	N/A	N/A	N/A	1	99	0	0	18.56 ± 0.22
Mix 1	0.1	N/A	N/A	99.9	N/A	N/A	15.99 ± 0.82	0	24.30 ± 0.64
Mix 2	1	N/A	N/A	99	N/A	N/A	14.15 ± 0.19	0	19.91 ± 0.36
Mix 3	10	N/A	N/A	90	N/A	N/A	13.80 ± 0.02	0	16.04 ± 0.44
Mix 4	30	N/A	N/A	70	N/A	N/A	13.78 ± 0.02	0	15.46 ± 0.83
Mix 5	70	N/A	N/A	30	N/A	N/A	13.63 ± 0.02	0	14.00 ± 0.13
Mix 6	90	N/A	N/A	10	N/A	N/A	13.57 ± 0.01	0	13.80 ± 0.09
Mix 7	99	N/A	N/A	1	N/A	N/A	13.58 ± 0.03	0	13.80 ± 0.08
Mix 8	99.9	N/A	N/A	0.1	N/A	N/A	13.57 ± 0.01	0	13.80 ± 0.04

Abbreviation: N/A, not applicable.

^a^ Data (average ± *SD*) represent 20 replicates.

The amplification plots of Sheep‐FAM are visible for mixtures in which the percentage of ovine meat was ≥10%, whereas being absent in mixtures where this percentage was of 1% and 0.1% (Figure 5a) (confidence limit: 95%). In addition, artificial binary mixtures containing ovine and caprine meat were used to evaluate the detectability of method. The percentages of ovine meat in the mixtures were 99.9%, 99%, 90%, 70%, 30%, 10%, 1%, and 0.1% (w/w). The corresponding percentages of caprine meat in the mixtures were 0.1%, 1%, 10%, 30%, 70%, 90%, 99%, and 99.9%. As shown in Figure 5b and Table 5, DNA Ct values increased with the decrease of the percentage of ovine meat in the mixtures (Figure 5b), with Ct = 0 in mixtures with a percentage of ovine meat of 1% and 0.1%. The amplification plots of Sheep‐FAM were visible in ≥10% ovine percentages, whereas being absent in mixtures with a ovine meat percentage of 1% and 0.1% (Figure 5b) (confidence limit: 95%). The Ct values of Goat‐HEX were increasing with the decreasing of caprine meat in the mixtures (Figure 5b), and the Ct values for 0.1% and 1% caprine percentages were 0. The amplification plots of Goat‐HEX was steadily appeared in ≥10% caprine percentages, not shown in the percentage of 0.1% and 1% (Figure 5b). The endogenous control (Control‐ROX) was steadily amplified in all eight mixtures (Figure 5a,b). Our data suggest that our triplex real‐time PCR with endogenous control can simultaneously detect ovine and caprine DNA meat mixtures.

In a similar set of experiments as before, we used binary dairy mixtures containing caprine and bovine milk to evaluate the efficiency of the triplex real‐time PCR. The percentages of caprine milk in the mixtures were 99%, 95%, 90%, 70%, 30%, 10%, 5%, and 1% (w/w). The corresponding percentages of bovine milk in the mixtures were 1%, 5%, 10%, 30%, 70%, 90%, 95%, and 99%. As shown in Figure 5c and Table 5, with the decreasing of caprine milk in the mixtures, the Ct values were increasing, and the Ct values for 5% and 1% caprine percentage were 0. The amplification plots of Goat‐HEX were steadily appeared in ≥10% caprine percentages, not shown in the percentage of 5% and 1% (Figure 5c) (confidence limit: 95%). Furthermore, the endogenous control (Control‐ROX) was steadily amplified in all eight dairy mixtures (Figure 5c). In conclusion, our data suggest that our newly developed triplex real‐time PCR method with an endogenous control can simultaneously detect caprine DNA in dairy mixtures.

As our results show, probe amplification is observed in mixtures where the corresponding meat percentage is at least of 10%, which in the context of meat adulteration in industry is common and profitable. Therefore, we propose that our PCR method can be used to certificate ovine and caprine meat and dairy products in mixtures. Still, the limit of detection of our method did not reach 1%–0.1% discrimination (average level), which might be due to our specific primer and probe design. In our triplex real‐time PCR method, competition between the three probes (Sheep‐FAM, Goat‐HEX, and Control‐ROX) for the template amplified by the shared primers is fundamental for simultaneous identification of ovine and caprine DNA and for monitoring the PCRs. The primer pair in this study is designed to target a conserved genomic region conserved across sheep, goat, cow, horse, and pork. We hypothesize that the competition of PCR resources in the triplex real‐time PCR for detection of ovine and caprine DNA from ovine/porcine, ovine/caprine meat mixtures, and caprine/bovine milk mixtures imposes restrictions on the limit of detection above 10%. Therefore, meat mixtures containing sheep and chicken were used to verify our hypothesis and examine the limit of detection of the method. As shown in Figure 5d, Sheep‐FAM amplification was detected in meat mixtures with ≥0.1% of ovine meat. The lower limit of detection in the sheep–chicken meat mixture indicates that methods based on species‐specific primer pairs are more sensitive in DNA identification in meat and dairy mixtures, compared to methods using species‐conserved primers. Currently, our team is developing two types of real‐time PCR systems for product authenticity certification. On the one hand, we developed species‐conserved primers and multichannel probes for high‐throughput evaluation. On the other hand, we also developed species‐specific primers and a probe for qualitative and quantitative analysis of low abundant contamination (adulteration).

## 4 CONCLUSIONS

The aim of this work was to develop a triplex *Taq*Man real‐time PCR for the simultaneous detection of ovine and caprine DNA in meat and dairy products and to include an endogenous control to avoid false‐negative results. The method is based on the design of species‐conserved primers, that is, a primer pair that targets a conserved genomic region across different species, an endogenous control probe, and species‐specific probes. Oligonucleotide design was optimized to ensure all oligonucleotides were compatible to be amplified in the same real‐time PCR. Our results show that our method allows the specific identification of ovine and caprine DNA, the limits of detection of 0.001 ng and 0.01 ng (ovine DNA in fresh and processed meat samples), and 0.00025 ng, 0.005 ng, and 0.01 ng (caprine DNA in fresh meat, milk, and cheese samples, respectively). Our triplex real‐time PCR method with endogenous control is, thus, a specific and highly sensitive technique for the certification of meat and dairy products derived from sheep and goat.

## CONFLICT OF INTEREST

All authors declared no conflict of interest.

## ETHICAL APPROVAL

This study does not involve any human or animal testing.

## Data Availability

Data available on request from the authors.
